# Influence of harvesting method on biomass yield and antimicrobial activity of *Isochrysis galbana* and *Phaeodactylum tricornutum* extracts

**DOI:** 10.1007/s10529-026-03741-5

**Published:** 2026-05-16

**Authors:** Edgard Freitas de Siqueira Junior, Luzimar Gonzaga Fernandez, Edson Delgado Rodrigues, Suzana Telles da Cunha Lima

**Affiliations:** 1 Institute of Biology, Federal University of Bahia, Salvador, Bahia Brazil; 2 Department of Life Sciences, State University of Bahia, Salvador, Bahia Brazil

**Keywords:** Bactericide, Bioactivity, Harvesting, Microalgae

## Abstract

Microalgae are recognized as promising sources of high-value bioactive compounds with applications in agricultural and pharmacological industries; however, conventional biomass harvesting methods may reduce yield, compromise bioactivity, and increase processing costs. This study aimed to evaluate the effect of electroflocculation on biomass recovery and the preservation of antimicrobial activity of microalgal extracts, in comparison with centrifugation. Cultures of both species were harvested using electroflocculation and centrifugation, followed by biomass quantification and extraction of bioactive compounds. The antibacterial activity of the extracts was assessed against bacterial strains of clinical and industrial relevance, allowing a comparative evaluation of harvesting efficiency and bioactivity preservation. Electroflocculation resulted in higher biomass recovery compared to centrifugation for both microalgal species. In addition, extracts obtained from electroflocculated biomass exhibited higher antibacterial activity under the tested conditions, indicating improved preservation of bioactive compounds during the harvesting process. These findings demonstrate that electroflocculation is a clean, efficient, and sustainable method for microalgal biomass harvesting, suggesting advantages over conventional centrifugation in terms of yield and maintenance of antimicrobial activity, and supporting its application in biotechnological processes aimed at producing bioactive compounds from microalgae.

**Supplementary Information:**

The online version contains supplementary material available at 10.1007/s10529-026-03741-5.

## Introduction

Microalgae are single-celled organisms in marine and freshwater environments, acting as primary producers and generating oxygen through photosynthesis. They produce valuable compounds, like phenolic acids, which are known for various biological activities (Kwak et al. 2014; Tan, et al. 2018; Andriopoulos et al. 2022). In aquaculture, they are a key food source, and their high protein content makes them a promising alternative for non-animal protein production (Wang, et al. 2021).

*Isochrysis galbana* Parke and *Phaeodactylum tricornutum* Bohlin are valuable biotechnologically for producing organic molecules. They are rich in polyunsaturated fatty acids (PUFAs) like EPA, DHA, and ARA, essential for human health, especially in infants (Adarme-vega et al. 2012; Crawford 2000). *I. galbana* also contains fucoxanthin, a highly nutritious compound with strong antioxidant properties (Kim et al. 2012; Blanco-llamero et al., 2021; Bustamam et al. 2021). However, obtaining microalgae biomass is one of the main obstacles to producing biocompounds at an industrial scale. It may generate a low biomass yield, which makes the separation process quite expensive. Adding organic solvents for flocculation is common, but the risk to human health and environmental impacts makes the cultivation unfeasible at industrial scale (Murador et al. 2019; Tan, et al., 2020).

Electroflocculation is an efficient, low-cost method for harvesting microalgae biomass without chemicals. It oxidizes the anode to produce a metallic flocculant that neutralizes cell charges, causing aggregates to form and rise to the surface (Tan, et al. 2018; Suparmaniam et al. 2019). The method is used for wastewater treatment and has been shown by our group to effectively serve as harvesting procedure, increasing biomass while preserving antioxidant activity (Ramos et al. 2017, 2020). This study aims to demonstrate the potential of electroflocculation to enhance algal biomass yields without compromising the bactericidal effect of ethanolic extracts against gram-positive and gram-negative strains.

## Materials and methods

### Microalgae, growing conditions, and maintenance

The marine microalgae used were *I. galbana* and *P. tricornutum*, obtained from the microalgae collection of the Biology Institute at the Federal University of Bahia (UFBA). Strains were registered at SISGEN (number A653955), the National System for Management of Genetic Heritage and Associated Traditional Knowledge (https://sisgen.gov.br), at the Botanical Research and Herbarium Management System (BRAHMS) platform and deposited at ALCB Herbarium from the Biology Institute of UFBA under IBL-C007 (*P. tricornutum*) and IBLC-013 (*I. galbana*) voucher numbers. Cultivation conditions included a 12 h photoperiod, luminosity of 35 μmol of photons m-2 s-1, temperature of 22 °C, pH 7.0, and constant aeration. Conway culture medium (Lourenço, 2006) was used for both species and for *P. tricornutum*, a diatom, 0.32 M Na2SiO3 was added. Microalgae were grown in a tubular photobioreactor, each strain in triplicate. Biomass growth was monitored daily using cell counting in the Neubauer chamber. The number of cells per mL (C) was determined according to the equation below:

### Harvesting methods

The biomass was harvested at the end of the stationary growth phase using two different methods: centrifugation (MPW-351®) at 5000 rpm for 45 min (repeated until all culture was used) and electroflocculation by a continuous current at 19 V or 95 W for 15 min, using aluminium electrodes (13, 14). Different electrode materials (including copper) and configurations (e.g., variations in thickness and perforation) were preliminarily evaluated, and aluminium was selected based on its performance and absence of visible residues in the culture medium. Afterward, the cells were lyophilized using a freeze dryer (Enterprise II, Terroni®) and kept at − 20 °C.

### Preparation of extracts

After thawing, 2 g of dry biomass from each strain was extracted with 100 mL of absolute ethyl alcohol, vortexed, and sonicated in two 6-min cycles at 10% power. The mixture was agitated for 72 h, centrifuged at 5000 rpm for 5 min, and the supernatant collected. This process was repeated until the biomass was decolorized. The extract was concentrated using a Vacuum Rotary Evaporator. The same procedure was applied to biomass obtained by electroflocculation.

### Microorganisms tested

Five standard strains of the American Type Culture Collection (ATCC) were used, being three of Gram-positive bacteria: *Staphylococcus aureus* ATCC 6598, *Bacillus subtilis* ATCC 6633, *Bacillus cereus* ATCC 0096; and two Gram-negative: *Escherichia coli* ATCC 94863, *Pseudomonas aeruginosa* ATCC 27853. All strains were obtained from the Tropical Culture Collection (CCT) of the André Tosello Foundation (https://fat.org.br) and are kept in the Laboratory of Exact and Earth Sciences I of State University of Bahia, Salvador, Bahia.

### Evaluation of antibacterial activity

The antibacterial activity of *I. galbana* and *P. tricornutum* extracts was assessed using the disc diffusion method, following the CLSI M2-A8 standard (Karaman et al 2003). The extracts were diluted in 1% DMSO to obtain 10 mg/mL solutions. A bacterial suspension of each microorganism was prepared to the 0.5 McFarland turbidity standard and inoculated onto Mueller–Hinton agar. Sterile filter paper discs were impregnated with 10 μL of the ethanol extract and placed on agar. Inhibition zones were measured after 24 h. Tests were conducted in triplicate, with Azithromycin (15 μg) and 1% DMSO (10 μL) as positive and negative controls, respectively.

Statistical analysis was performed using one-way ANOVA followed by Tukey’s post hoc test, with significance set at p < 0.05.

## Results and discussion

The extracts produced from biomass of both species tested, obtained by centrifugation and electroflocculation, showed activity mainly against Gram-positive bacteria, this being the first report of antibacterial activity of *I. galbana* microalgae against *B. cereus*. Only *P. tricornutum* demonstrated activity against a gram-negative strain, *E. coli*, and just for the centrifuged method. Table 1 shows the results for the ethanolic extracts produced from both harvesting methods. Electroflocculation produced biomass whose extracts showed a higher percentage of inhibition biomass which extracts have higher percentage of inhibition (44–65%) compared to that obtained by the centrifugation method (21.43–40%). For *S. aureus*, it showed nearly twice the antibacterial activity of extracts. This result is significant as it highlights the influence of the harvesting method on the preservation of bioactive compounds. Electroflocculation appears to better maintain antimicrobial activity compared to centrifugation, suggesting reduced degradation of active molecules during biomass recovery. The observed activity against clinically relevant bacteria further supports the potential application of this approach. Harvesting cells from indoor cultivation is a costly step, often hindering the scalable production of algae products (Tripathi & Kumar, 2017). In this study, the method not only increased biomass yield but also enhanced the bactericidal activity of the extracts, which could be valuable for edible and pharmacological applications by preserving the bioactivity of target molecules.

**Table 1:** Antimicrobial activity of ethanolic extracts of *I. galbana* and *P. tricornutum* produced from biomass obtained by centrifugation and electroflocculation

Microalgae	Bacteria	Control + (mm)	Centrifugation IH (mm)	IH (%)	Electroflocculation IH (mm)	IH (%)
*I. galbana*	*S. aureu*s	26	9 ± 1.2^a^	38.46	—	—
	*B. subtilis*	25	10 ± 1.2^a^	40.00	11 ± 1.2^a^	44.00
	*B. cereus*	24	—	—	15 ± 1.2^b^	62.50
	*E. coli*	28	—	—	—	—
	*P. aeruginosa*	23	—	—	—	—
*P. tricornutum*	*S. aureu*s	26	9 ± 0.7^a^	34.65	17 ± 0.9^b^	65.38
	*B. subtilis*	25	8 ± 1.2^a^	32.00	12 ± 1.8^b^	48.00
	*B. cereus*	24	9 ± 0.9^a^	37.57	15 ± 1.6^b^	62.50
	*E. coli*	28	6 ± 1.0^a^	21.43	—	—
	*P. aeruginosa*	23	—	—	—	—

Values are expressed as mean ± standard deviation (n = 3). Different letters in the same row indicate statistically significant differences between harvesting methods (p < 0.05), according to one-way ANOVA followed by Tukey’s test. Percentages were calculated in relation to the inhibition halo of the positive control (Azithromycin 15 µg)

*IH* inhibition halo

Antimicrobial activity is linked to unsaturated fatty acids. Alsenani et al. (2020) characterized the ethanolic extracts of *I. galbana* using Mass Spectrometry and NMR, identifying pheophytin A, docosahexaenoic acid, oleic acid, and linoleic acid. Their antimicrobial evaluation showed that *I. galbana* extract effectively inhibited *Listeria monocytogenes*, *S. aureus*, and *B. subtilis*, with inhibition zones of 19–20 mm. The mechanism involved in the antimicrobial effect of polyunsaturated fatty acids was studied by Zheng et al. (2005) and Sado-Kamdem et al. (2009). The authors identified that these molecules impair bacterial lipid metabolism through enzymatic inhibition.

He et al. (2019) found a significant amount of eicosapentaenoic acid in *P. tricornutum* biomass, and Desbois et al. (2008) demonstrated its ability to inhibit multidrug-resistant *S. aureus*. Wang et al. (2021) reported a minimum inhibitory concentration of 18 μg/mL for eicosapentaenoic acid against *Vibrio* spp., suggesting promising antimicrobial potential. However, it showed limited activity against *E. coli* and *P. aeruginosa*. Gram-negative bacteria are more difficult to treat due to their additional membrane, rich in saturated fatty acids, which restricts the diffusion of antimicrobial agents. While efficient, centrifugation is time-consuming, especially for small cells, and energy intensive. Additionally, it reduces biomass yield by exposing cells to shear forces, which causes cell damage and decreases the availability of bioactive compounds (Ramos et al. 2020).

The electroflocculation method for biomass harvesting, combined with bioactivity analysis of microalgae extracts, shows great promise but requires further investigation. Our team contributed significantly to this field, demonstrating biomass increases of up to four times for *P. tricornutum* and ten times for *I. galbana* (Ramos et al. 2017, 2020). However, the method showed slightly reduced antioxidant activity compared to centrifugation. Davoodbasha et al. (2021) found that electroflocculation achieved 95% efficiency for *Chlorella pyrenoidosa*, second only to FeCl₃ flocculation (98.5%), but the latter imparts a brown color to the medium, complicating pigment extraction. Additionally, Davoodbasha et al. (2021) reported that the use of salts for harvesting may increase costs. Our study found that electroflocculation enhanced the bactericidal activity of ethanol extracts from marine microalgae *I. galbana* and *P. tricornutum*. This technique yielded biomass effective against Gram-positive bacteria, including *Staphylococcus aureus*, which is clinically significant. As antibiotic resistance rises, microalgae offer a promising source of new compounds, with electroflocculation providing a cost-effective and non-toxic harvesting method. While the results provide a comparative assessment under standardized conditions, the antimicrobial activity was evaluated at a single extract concentration, which limits a more detailed quantitative comparison between harvesting methods. Future studies should include dose–response analyses and determination of minimum inhibitory concentrations (MIC) to further characterize the antimicrobial potential of the extracts. These findings highlight the potential of electroflocculation as a biomass harvesting method with advantages in preserving antimicrobial activity, supporting its continued investigation for applications in the pharmaceutical and food industries.

## Supplementary Information

Below is the link to the electronic supplementary material.

Supplementary file1 (JPG 632 KB)

## Data Availability

All data supporting the findings of this study are included in the article.
