# Supplementary figures and images for: Influence of harvesting method on biomass yield and antimicrobial activity of Isochrysis galbana and Phaeodactylum tricornutum extracts
Source: Biotechnol Lett. 2026 May 16;48(3):70. doi: 10.1007/s10529-026-03741-5 (PMC13179900; doi:10.1007/s10529-026-03741-5)

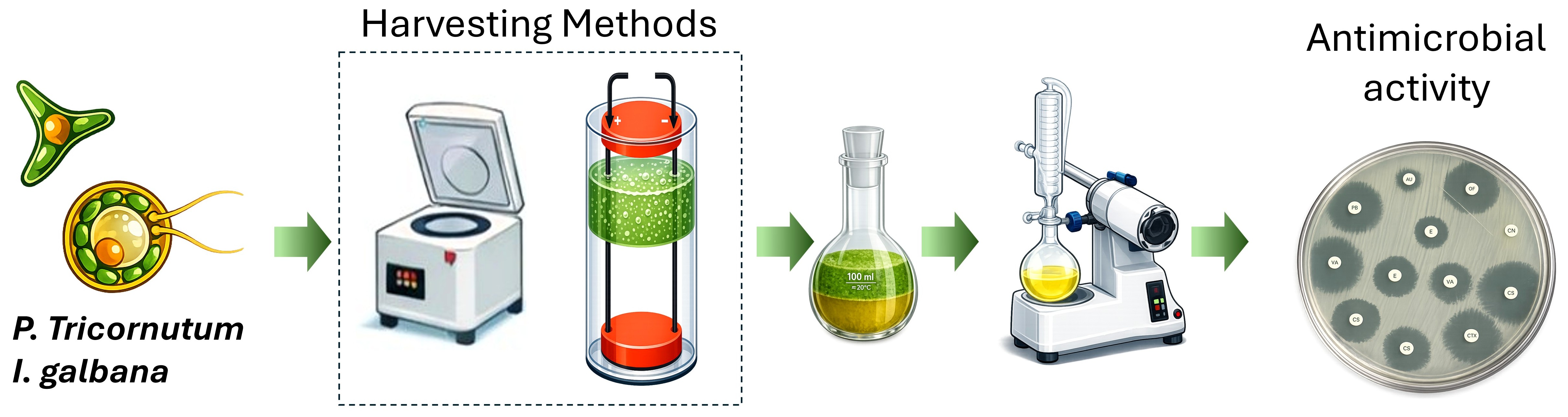

Supplement: Supplementary file 1 — Supplementary file1 (JPG 632 KB) [file 10529_2026_3741_MOESM1_ESM.jpg]
